# Adaptation to high salt concentrations in halotolerant/halophilic fungi: a molecular perspective

**DOI:** 10.3389/fmicb.2014.00199

**Published:** 2014-05-05

**Authors:** Ana Plemenitaš, Metka Lenassi, Tilen Konte, Anja Kejžar, Janja Zajc, Cene Gostinčar, Nina Gunde-Cimerman

**Affiliations:** ^1^Faculty of Medicine, Institute of Biochemistry, University of LjubljanaLjubljana, Slovenia; ^2^Biology Department, Biotechnical Faculty, University of LjubljanaLjubljana, Slovenia; ^3^Department of Biotechnology and Systems Biology, National Institute of BiologyLjubljana, Slovenia; ^4^Centre of Excellence for Integrated Approaches in Chemistry and Biology of Proteins (CIPKeBiP)Ljubljana, Slovenia

**Keywords:** halophilic/halotolerant fungi, *Hortaea werneckii* genome, *Wallemia ichthyophaga* genome, HOG signaling pathway, ion homeostasis

## Abstract

Molecular studies of salt tolerance of eukaryotic microorganisms have until recently been limited to the baker's yeast *Saccharomyces cerevisiae* and a few other moderately halotolerant yeast. Discovery of the extremely halotolerant and adaptable fungus *Hortaea werneckii* and the obligate halophile *Wallemia ichthyophaga* introduced two new model organisms into studies on the mechanisms of salt tolerance in eukaryotes. *H. werneckii* is unique in its adaptability to fluctuations in salt concentrations, as it can grow without NaCl as well as in the presence of up to 5 M NaCl. On the other hand, *W. ichthyophaga* requires at least 1.5 M NaCl for growth, but also grows in up to 5 M NaCl. Our studies have revealed the novel and intricate molecular mechanisms used by these fungi to combat high salt concentrations, which differ in many aspects between the extremely halotolerant *H. werneckii* and the halophilic *W. ichthyophaga*. Specifically, the high osmolarity glycerol signaling pathway that is important for sensing and responding to increased salt concentrations is here compared between *H. werneckii* and *W. ichthyophaga*. In both of these fungi, the key signaling components are conserved, but there are structural and regulation differences between these pathways in *H. werneckii* and *W. ichthyophaga*. We also address differences that have been revealed from analysis of their newly sequenced genomes. The most striking characteristics associated with *H. werneckii* are the large genetic redundancy, the expansion of genes encoding metal cation transporters, and a relatively recent whole genome duplication. In contrast, the genome of *W. ichthyophaga* is very compact, as only 4884 protein-coding genes are predicted, which cover almost three quarters of the sequence. Importantly, there has been a significant increase in their hydrophobins, cell-wall proteins that have multiple cellular functions.

## Introducing *Hortaea werneckii* and *Wallemia ichthyophaga*

Studies of fungal populations in hypersaline environments have revealed the high diversity of fungal species (Gunde-Cimerman et al., 2000), most of which do not require salt for growth, and have their growth optimum in the absence of salt. The dominant fungal group in the hypersaline waters of salterns are the melanized polymorphic black yeast, the most abundant and adapted species of which is *Hortaea werneckii*. *H. werneckii* is naturally adapted to fluctuating salt concentrations in its environment, and it can grow without salt and in up to saturated NaCl. Its optimum for growth is between 0.8 M and 1.7 M NaCl. Another successful survivor in these extremely salty environments is the basidiomycetous fungus *Wallemia ichthyophaga* (Zalar et al., 2005b), which does not grow without salt, and is therefore obligately halophilic.

Due to their different ecology and halotolerances, these two fungi represent highly relevant organisms for the study of eukaryotic adaptation to life at high salt. Studies of haloadaptation mechanisms of *H. werneckii* started some 15 years ago (for reviews, see Petrovič et al., 2002; Gunde-Cimerman and Plemenitaš, 2006; Plemenitaš et al., 2008; Gostinčar et al., 2011), while with *W. ichthyophaga*, these studies began later, and are thus less advanced.

## The extremely halotolerant *Hortaea werneckii*

*H. werneckii* (Horta) Nishim and Miyaji (Capnodiales, Dothideomycetes) is a melanized yeast-like ascomycete that is known as the causative agent of *tinea nigra*, a superficial mycotic infection of the human palm (de Hoog and Gerrits van den Ende, 1992). *H. werneckii* has been isolated from diverse environments with low water activity (a_w_), including salty food (Mok et al., 1981), seawater (Iwatsu and Udagawa, 1988), beach soil (de Hoog and Guého, 1998), rocks (Stanley et al., 1982), wood immersed in hypersaline waters (Wollenzien et al., 1995; Zalar et al., 2005a) and microbial mats (Cantrell et al., 2006). However, it appears that its primary habitat is hypersaline water in the evaporate ponds of solar eutrophic salterns (Gunde-Cimerman et al., 2000).

While *H. werneckii* has been extensively described in our earlier review papers (Petrovič et al., 2002; Gunde-Cimerman and Plemenitaš, 2006; Plemenitaš et al., 2008; Gostinčar et al., 2011), *W. ichthyophaga* has not been reviewed to date, and thus it is presented below in more detail.

### The halophilic *Wallemia ichthyophaga*

*Wallemia* Johan-Olsen (Wallemiales, Wallemiomycetes) is a genus of cosmopolitan xerophilic fungi that can be found in a wide variety of environments that are characterized by low a_w_ (Samson et al., 2004; Zalar et al., 2005b). Its phylogenetic position was unclear until recently, and previously it has been placed in various positions in the Basidiomycota phylogenetic tree, from the root of basidiomycetes (Zalar et al., 2005b), to *incertae sedis* (Hibbett et al., 2007), to being a sister group of the Agaricomycotina and Ustilaginomycotina (Matheny et al., 2006). Genome sequencing has shown that it is indeed a sister group of the Agaricomycotina (Padamsee et al., 2012; Zajc et al., 2013). The Wallemiomycetes split from the Agaricomycotina ancestors an estimated 250 million years ago (Zajc et al., 2013). Initially the genus contained only one species, but it was later segregated into three species based on differences in conidial size, xerotolerance, and sequence data: *W. ichthyophaga*, *Wallemia sebi* and *Wallemia muriae* (Zalar et al., 2005b). To date, only around 20 strains of *W. ichthyophaga* have been isolated from hypersaline waters of solar salterns, bitterns (i.e., magnesium-rich residual solutions in salt production from sea water) and salted meat (Zalar et al., 2005b). In addition to phylogenetic differences, *W. ichthyophaga* is also distinguished from the other two representatives of this genus by its characteristic morphology and halophilic physiology (Zalar et al., 2005b; Kralj Kunčič et al., 2010).

Although xerotolerance is rare in the Basidiomycota, all three *Wallemia* spp. are among the most xerophilic fungal taxa known to date (Zalar et al., 2005b). However, while *W. sebi* and *W. muriae* strongly prefer high concentrations of non-ionic solutes over those of NaCl (Kralj Kunčič et al., 2013), the opposite is true for *W. ichthyophaga* (Zalar et al., 2005b). For growth, *W. ichthyophaga* requires at least 1.5 M NaCl, or some other osmolyte at an equivalent a_w_. Such a narrow ecological amplitude of salt concentrations is common for specialized archaeal halophiles, but it is exceptional in the fungal kingdom. Hence, *W. ichthyophaga* is a rare fungal example of an obligate extremophilic specialist (Gostinčar et al., 2010), and it is considered to be the most halophilic fungus known to date. Although it even thrives in saturated NaCl solution, its *in vitro* growth optimum is between 2.6 M and 3.5 M NaCl, which is the highest described among fungi (Zajc et al., 2014). It also tolerates high concentrations of salts other than NaCl; e.g., MgCl_2_ (our unpublished data).

## Sensing hyperosmolarity in *H. werneckii* and *W. ichthyophaga*

Exposure to high salinity includes two different environmental stimuli for the cell: osmotic stress, and ionic stress. In general, hyperosmotic stress in non-adapted organisms causes immediate water efflux from the cell, which reduces the turgor pressure and triggers cytosol dehydration, thereby increasing the concentrations of the solutes in the cytoplasm (Petelenz-Kurdziel et al., 2011). In particular, under high ionic stress conditions, ions (e.g., Na^+^) enter the cell, which leads to increased intracellular ion concentrations, which subsequently damage the membranes as well as the cytosolic systems. The main survival strategies to counteract changes in turgor pressure for fungi that are adapted to life at low a_w_ are an accumulation of compatible solutes that do not interfere with vital cellular protein functions, and maintenance of intracellular concentrations of Na^+^ below toxic levels (Blomberg and Adler, 1992). Both *H. werneckii* and *W. ichthyophaga* use the strategy of compatible organic solutes to maintain low intracellular Na^+^ concentrations, with glycerol being the main solute used.

The main signaling pathway in fungi that is responsible for cellular stress responses is the high osmolarity glycerol (HOG) pathway, which has been extensively studied in the context of osmotic stress in *S. cerevisiae*. The production and homeostasis of the compatible solute glycerol is one of the main targets under the control of this signaling pathway (Hohmann et al., 2007). The core of the pathway is represented by the mitogen-activated protein kinase (MAPK) signaling module, which is known for its high evolutionary conservation and its activation by sequential phosphorylations (Widmann et al., 1999). This upstream part of the HOG pathway consists of two branches, which are functionally redundant but structurally distinct. They are known as the SHO1 and SLN1 branches and they converge at the MAPK kinase (MAPKK) Pbs2. Upon hyperosmotic shock, when the cell loses some of its water, the MAPK Hog1 is phosphorylated and activated by the upstream MAPKK Pbs2. The main effect of this HOG pathway activation is glycerol production, which restores the cellular osmotic balance. When turgor is re-established, Hog1 is dephosphorylated by phosphatases (Saito and Posas, 2012).

### The HOG signal transduction in *H. werneckii* and *W. ichthyophaga*

In *H. werneckii* and *W. ichthyophaga*, several components of the HOG pathway have been identified and characterized (Lenassi and Plemenitas, 2007; Fettich et al., 2011; Konte and Plemenitaš, 2013). Since their sequenced genomes became available, the presence of some novel HOG components has been confirmed through homology searches. Altogether, there are many similarities between the HOG pathways in *H. werneckii*, *W. ichthyophaga*, and *S. cerevisiae*, although there are also some important differences that might explain the different halotolerant/halophilic characters of *H. werneckii* and *W. ichthyophaga*.

**Figure 1:**
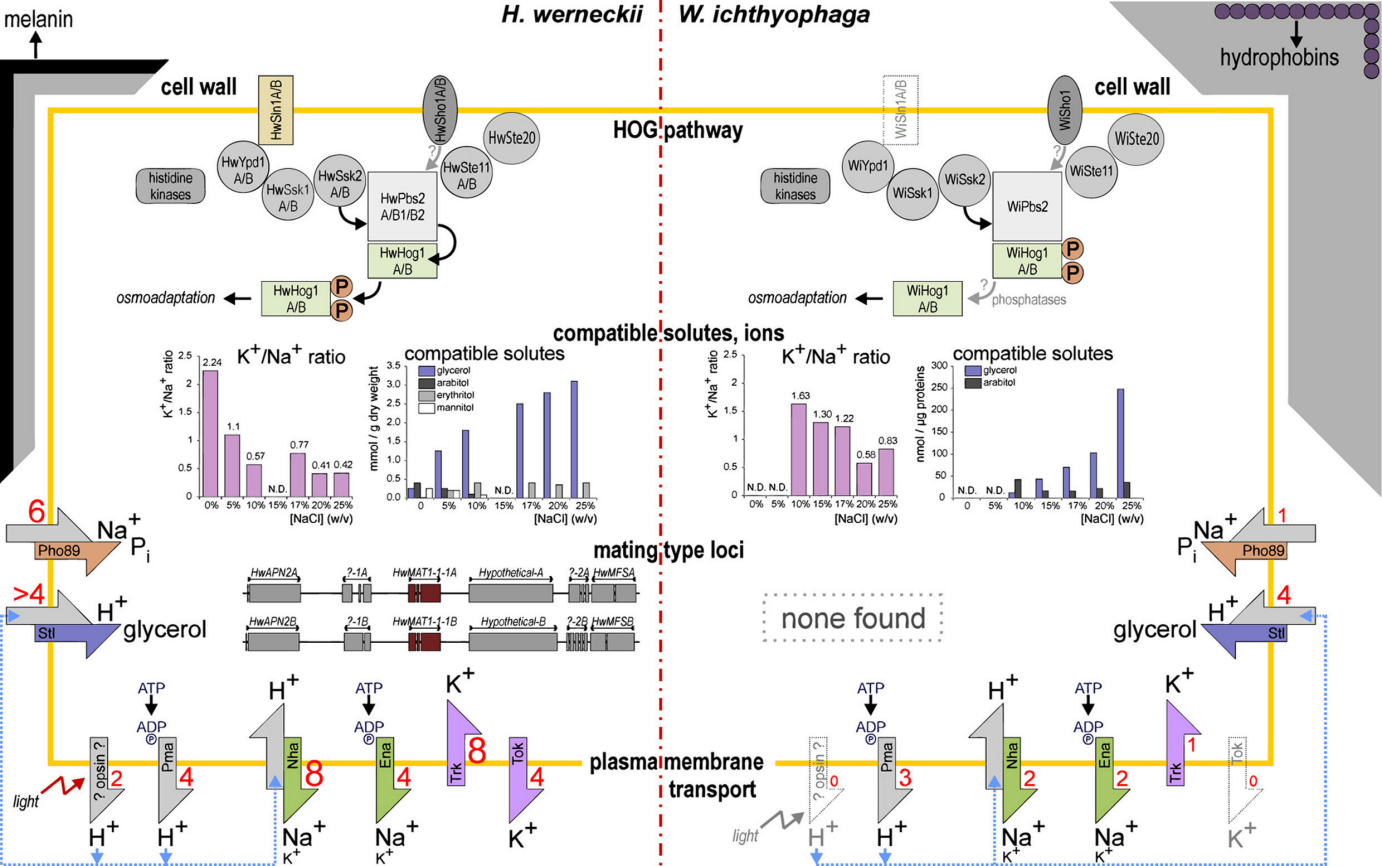
**Model of the key adaptations in *H. werneckii* and *W. ichthyophaga***.

The presence of homologs of Sho1, Ste20, and Ste11 has been confirmed for the genomes of *H. werneckii* and *W. ichthyophaga*, with two copies of each component in *H. werneckii* (Figure 1; Lenassi et al., 2013; Zajc et al., 2013; our unpublished data). Two isoforms of the *H. werneckii* putative osmosensor protein HwSho1A and HwSho1B fully complement the function of the homologous *S. cerevisiae* Sho1 protein and they can activate the HOG pathway under osmotic stress in *S. cerevisiae*. Structurally, when compared to other fungal Sho1 homologs, they contain a conserved SH3 domain and a divergent Ste11-binding motif (Fettich et al., 2011). On the other hand, the SH3 domain of *W. ichthyophaga* (Wi)Sho1 is functional when it is attached to the N-terminal part of *S. cerevisiae* Sho1, although the whole sequence of WiSho1 does not appear to function correctly in *S. cerevisiae* (our unpublished data). Regardless of the complementation of the HwSho1 protein and the WiSho1 SH3 domain, data from recent preliminary investigations addressing the role of the SHO1 branch pathway in osmo-adaptation do not support the involvement of this branch in the signal transfer downstream to heterologously expressed HwPbs2 and WiPbs2 in *S. cerevisiae*. WiSte11 also failed to complement ScSte11 in *S. cerevisiae ste11*Δ*ssk2ssk22*Δ cells, further supporting this hypothesis (our unpublished data).

The other branch of the HOG pathway, which was named as SLN1 after the transmembrane Sln1 hybrid histidine kinase, transmits its signals via a Sln1–Ypd1–Ssk1 phosphorelay. Sln1 kinase is inactive under hyperosmolar conditions, where Ssk1 is dephosphorylated and therefore binds to the autoinhibitory region of Ssk2 and Ssk22, which triggers their autophosphorylation (Saito and Posas, 2012). The *H. werneckii* histidine kinases HwHhk7A and HwHhk7B have been identified and characterized in more detail (Figure 1; Lenassi and Plemenitas, 2007). HwHhk7A and HwHhk7B lack the transmembrane domain, but otherwise they have a typical eukaryotic hybrid histidine-kinase-domain composition. Their transcription in *H. werneckii* depends on the extracellular salt concentration, and when they are expressed in *S. cerevisiae*, they increase its osmotolerance (Lenassi and Plemenitas, 2007). Both the *H. werneckii* and *W. ichthyophaga* genomes also contain homologs of the group III histidine kinases. This group of cytosolic histidine kinases can act as osmosensors through their HAMP domain repeats (Meena et al., 2010). On the other hand, the membrane-spanning Sln1-like histidine kinase is present in the genome of *H. werneckii*, again in two copies, but there is no evidence for it in the genome of *W. ichthyophaga* (Figure 1), which suggests that the group III histidine kinases are probably involved in osmosensing in this halophilic fungus. Of the other proteins involved in the SLN1 branch, two forms of each of the *S. cerevisiae* homologs have been found in the genome of *H. werneckii*, HwYpd1A/B, HwSsk1A/B, and two homologs of the MAPKK kinase Ssk2, HwSsk2A/B (Table 2) (Lenassi et al., 2013; our unpublished data). Also *W. ichthyophaga* has the proteins WiYpd1, WiSsk1, and WiSsk2 (Figure 1, Table 2; Zajc et al., 2013; our unpublished data).

The signals from both branches of the HOG pathway in *S. cerevisiae* converge at the MAPKK Pbs2 scaffold, which transmits the signals further to Hog1 (Saito and Posas, 2012). Two gene copies of the MAPKK HwPbs2 have been identified in *H. werneckii* and one in *W. ichthyophaga*, WiPbs2 (Figure 1; Lenassi et al., 2013; Zajc et al., 2013; our unpublished data). However, preliminary data show that the kinases HwPbs2 and WiPbs2 do not interact with the *S. cerevisiae* Sho1 protein (our unpublished data). This suggests that the SHO1 branch is not involved in HOG pathway activation in *H. werneckii* and *W. ichthyophaga*.

We had previously identified and characterized only one isoform of the final MAPK HwHog1 (Turk and Plemenitaš, 2002; Lenassi et al., 2007); however, the *H. werneckii* whole genome sequence revealed another copy of the *HwHOG1* gene, the characterization of which is currently in progress. We have also identified two Hog1-like kinase paralogs in *W. ichthyophaga*, although all of the other HOG pathway components are represented by only single gene copies (Figure 1; Konte and Plemenitaš, 2013).

HwHog1A, HwHog1B, WiHog1A, and WiHog1B are all considerably shorter than ScHog1, although they contain the conserved domains and motifs that are characteristic of the MAPKs, such as the ATP-binding region, Asp in the active site, a TGY phosphorylation motif, a common docking domain, and a Pbs2-binding domain. While HwHog1A, HwHog1B, and WiHog1B are fully functional kinases in the *S. cerevisiae hog1*Δ background (Lenassi et al., 2007; Konte and Plemenitaš, 2013; our unpublished data), WiHog1A can only partly restore the osmotolerance of the *hog1*Δ strain. Lower phosphorylation levels, lower *GPD1* induction, and greater cross-talk with the mating pathway indicate that WiHog1A cannot interact optimally with the protein partners in *S. cerevisiae*. WiHog1B, on the other hand, is a fully functional kinase in the *S. cerevisiae hog1*Δ background. Moreover, WiHog1B even improves the salt tolerance of *S. cerevisiae* (Konte and Plemenitaš, 2013). We have also demonstrated that in contrast to *S. cerevisiae*, where the levels of *HOG1* mRNA remain unchanged when the cells are exposed to osmotic shock (Brewster et al., 1993), the transcript levels of *HOG1*-like kinases in *H. werneckii* and *W. ichthyophaga* are salt-dependent (Lenassi et al., 2007; Konte and Plemenitaš, 2013).

Upon hyperosmotic shock in *S. cerevisiae*, Hog1 is rapidly phosphorylated and it translocates into the nucleus. After the cell adapts to the higher osmolarity, Hog1 is dephosphorylated by phosphatases in a negative-feedback manner (Hohmann et al., 2007). Phosphorylation patterns in the extremely halotolerant *H. werneckii* and the obligate halophile *W. ichthyophaga* appear to be more complex. In *H. werneckii*, we have observed a phosphorylation mechanism that is similar to that of *S. cerevisiae*, although the HwHog1 kinase is noticeably phosphorylated only when the *H. werneckii* cells were exposed to ≥3 M NaCl (Turk and Plemenitaš, 2002). While in *S. cerevisiae* constitutive Hog1 phosphorylation is lethal (Maeda et al., 1994), in *W. ichthyophaga* this is not the case. Even more interestingly, *W. ichthyophaga* has a completely “opposite” phosphorylation pattern to that of *S. cerevisiae*: WiHog1 kinase is dephosphorylated after hypo-osmotic or hyperosmotic shock in *W. ichthyophaga*, and it is constitutively phosphorylated under optimal osmotic conditions (3.4 M NaCl). These data indicate an important role for the phosphatases in the regulation of the HOG pathway in *W. ichthyophaga* (Konte and Plemenitaš, 2013). This model has already been reported for *Cryptococcus neoformans*, where some serotypes show inverted *W. ichthyophaga*-like phosphorylation patterns (Bahn et al., 2007).

When activated, HwHog1 is translocated into the nucleus, where it associates with the chromatin of osmoresponsive genes and induces or represses their expression (Vaupotič and Plemenitaš, 2007). A transcriptional response to hyperosmolar stress of 95 differentially expressed genes has been reported for the comparison of moderately (3 M NaCl) and extremely (4.5 M) osmolar environments. Data from the ChIP method show that 36 of these genes physically interact with HwHog1 in long-term adaptation to extreme environments (Vaupotič and Plemenitaš, 2007). In 17 out of these 36 genes, simultaneous co-localization of RNA polymerase II was seen. More than half of differentially expressed genes are related to general metabolism and energy production, and the other osmoresponsive genes are involved in the biogenesis of mitochondria, protein biosynthesis, protein quality control, transport facilitation, the cell cycle, and the cell wall (Vaupotič and Plemenitaš, 2007). Thirteen of these 95 genes could not be classified. Certain osmoresponsive genes controlled by MAPK HwHog1 have been studied in greater detail. Genes that code for the P-type ATPases HwEna1 and HwEna2 are the *S. cerevisiae ENA1* homologs, and therefore they are believed to be involved in the maintenance of a low intracellular K^+^/Na^+^ ratio. Their transcription is salt regulated (Gorjan and Plemenitaš, 2006). Two homologs of the *S. cerevisiae* key enzyme in glycerol biosynthesis, the glycerol-3-phosphate dehydrogenase Gpd1, have been characterized in *H. werneckii*. These both show similar transcription profiles in response to different salt concentrations (Lenassi et al., 2011). We have also demonstrated that the MAPK WiHog1 can up-regulate the transcription of *GPD1* in *S. cerevisiae*. The regulation of other osmoresponsive genes that are potential targets of WiHog1 remains to be defined.

## *Hortaea werneckii* genome analysis

### Whole-genome duplication

The genome of *H. werneckii* was recently sequenced and it has been deposited at DDBJ/EMBL/GenBank under the accession number AIJO00000000 (Lenassi et al., 2013). The genome statistics are summarized in Table 1. The genome of *H. werneckii* has a size of 51.6 Mb, which is relatively large. In species belonging to the same order as *H. werneckii* (*Capnodiales*), the genome sizes are very variable, as they range from 21.88 to 74.12 Mb. The larger genome sizes are mostly due to a substantial amount of repetitive sequences. However, in *H. werneckii*, despite its large genome size, the proportion of repetitive sequences is only 1.02%. On the other hand, it contains 23,333 predicted genes, which is twice as many as the average number of predicted genes in other related fungi (approx. 11,955 genes) (Ohm et al., 2012). This large number of genes can be attributed to a relatively recent whole genome duplication, which resulted in two nearly identical copies of almost every protein of *H. werneckii* (Lenassi et al., 2013). This discovery is in line with our previous studies of several individual genes from *H. werneckii* that were present in two copies (Gorjan and Plemenitaš, 2006; Lenassi and Plemenitas, 2007; Fettich et al., 2011). In most cases, the expression of both of the gene copies is salt dependent, although their expression profiles differ (Lenassi and Plemenitas, 2007). It may well be that as a consequence of this whole genome duplication, *H. werneckii* can benefit from the potential advantages of large genetic redundancy, even though it is formally in a haploid stage (i.e., it is not a diploid that has resulted from the mating of two strains with opposite mating types, which would regain the haploid stage with meiosis before the next mating event; see below).

**Table 1:** **Genome statistics for *W. ichthyophaga* and *H. werneckii* (after Lenassi et al., 2013; Zajc et al., 2013)**.

**Statistic**	**Wi**	**Hw**
Coverage	>270×	~70×
Genome assembly size (Mbp)	9.63	51.6
Number of contigs	101	~20,000
Number of scaffolds	82	/
Gene models (n)	4884	23333
GC content (%)	45%	54%
GC content of exons (%)	48%	56%
Repeat content (%)	1.67%	1.02%

### Mating genes

The *H. werneckii* genome sequence has offered the opportunity to gain insight into the genetic information on the mating type(s) and on the mating strategy. To date, no sexual cycle has been described for *H. werneckii*. Using *M. graminicola* proteins that contain the alpha1 domain (Mat1-1-1) and the HMG domain (Mat1-1-2), we identified the putative *HwMAT1-1-1A* and *HwMAT1-1-1B* genes (Figure 1, Table 2), both of which are translated into 358 amino-acid proteins that contain the alpha1 domain and have an overall amino-acid sequence identity of 87.5% (Lenassi et al., 2013). Importantly, no homologs of the HMG-domain-containing Mat1-1-2 protein were found in *H. werneckii*, which indicates that this species is heterothallic, and that if it can still undergo sexual reproduction, this requires a strain that codes for the opposite mating type (in the case of the sequenced strain, this would be a strain with a Mat1-1-2 homolog; Lenassi et al., 2013).

**Table 2:** **Major proteins, identified in *H. werneckii* and *W. ichthyophaga* and presumably involved in adaptation mechanisms to increased salinity**.

**Description**	S. cerevisiae^*^	H. werneckii^*^	W. ichthyophaga^*^
**HOG PATHWAY**			
transmembrane osmosensor	Sho1	HwSho1A/B	
MAP kinase kinase	Pbs2	HwPbs2A/B	WiPbs2
MAP kinase	Hog1 (ScHog1)	HwHog1A/B	WiHog1A/B
MAP kinase kinase kinase	Ste11	HwSte11A/B	WiSte11
phosphorelay response regulator	Ssk1	HwSsk1A/B	WiSsk1
MAP kinase kinase kinase	Ssk2	HwSsk2A/B	WiSsk2
hybrid hystidine kinase		HwHhk7A/B	
tyrosine (Y) phosphatase dependent	Ypd1	HwYpd1A/B	WiYpd1
**MATING**			
mating-type protein	Mat1-1-1 or Mat1-2-1	HwMat1-1-1A/B	none
**COMPATIBLE SOLUTE MANAGEMENT**			
glycerol-3-phosphate dehydrogenase	Gpd1, Gpd2	HwGpd1A/B, HwGpd2A/B	WiGpd1, WiGpd2
glycerol-3-phosphatase	Gpp1, Gpp2		WiGpp1
glycerol proton symporter, plasma membrane	Stl1		4
aquaglyceroporin	Fps1		3
D-arabinitol-2-dehydrogenase			2
**ALKALI METAL ION CONCENTRATION MANAGEMENT**			
K^+^ efflux antiporter, plasma membrane	Nha1	8	2
K^+^ efflux channel, plasma membrane	Tok1	4	none
K^+^ uptake uniporter, plasma membrane	Trk1,2	8	1
Na^+^ (and Li^+^) efflux P-type ATPase, plasma membrane	Ena1-5	4	2
Na^+^/P_*i*_ symporter, plasma membrane	Pho89	6	1
H^+^ exporter P-type ATPase, plasma membrane	Pma1	4	3
K^+^/H^+^ antiporter, Golgi apparatus	Kha1	2	2
Na^+^/H^+^ antiporter, late endosomes	Nhx1	2	1
Na^+^, K^+^/H^+^ antiporter, vacuole	Vnx1	8	1
H^+^ V-type ATPase, subunit A	Vma1	2	1
K^+^/H^+^ antiporter, mitochondria	Mrs7/Mdm38	2	1

* *The columns contain protein names or number of homologs of each protein*.

### Alkali-cation transport systems

Eukaryotic microorganisms have developed numerous plasma-membrane transport systems to maintain their appropriate alkali cation levels, and in particular, to eliminate any surplus of toxic Na^+^ ions. The alkali-cation transport systems in *S. cerevisiae* and in non-conventional yeast have recently been reviewed (Arino et al., 2010; Ramos et al., 2011).

In *S. cerevisiae*, the plasma-membrane transporters Trk1 and Trk2 for K^+^ uptake, the Tok1 K^+^ channel, the Pho98 inorganic phosphate (P_*i*_)-Na^+^ symporter, the Ena Na^+^-ATPases, and the Nha1 Na^+^/H^+^ antiporter have all been well characterized (Arino et al., 2010). Together with these, non-specific protein transporters (e.g., Pm3, Qdr2) have been described to be involved in K^+^/Na^+^ fluxes across the plasma membrane (Arino et al., 2010). Trk transporters for K^+^ uptake, Nha antiporters, Ena ATPases, and Tok1 channels have also been identified in non-conventional yeast, together with the Hak K^+^/H^+^ symporters and the rare K^+^/Na^+^-uptake ATPase Acu (Ramos et al., 2011).

Physiological studies have shown that *H. werneckii* maintains very low intracellular K^+^ and Na^+^ levels (Kogej et al., 2005), even when it grows in the presence of 4.5 M NaCl, which suggested that it can effectively extrude Na^+^ ions and also prevent their influx. Analysis of *H. werneckii* genome has revealed considerable expansion of families of genes that encode plasma-membrane metal cation transporters, as presented schematically in Figure 1 and summarized in Table 2 (Lenassi et al., 2013). We identified eight homologs of the Trk1 and Trk2 K^+^ channels, with each containing the conserved TrkH domain that is typical for cation transport proteins. In general, they show low homology to the Trk1 protein, but the amino-acid sequence identity increases in the TrkH domain. We also identified four homologs of the Tok1 K^+^ channels, each of which contains two conserved transmembrane helices that are typical of this ion-channel family. Again, the homology to the Tok1 protein is low, but the identity is high in the transmembrane helices. The presence of eight homologs of the Nha1 Na^+^/K^+^, H^+^ antiporters was demonstrated, each of which contains a transmembrane region at the N-terminal, which is conserved through the Na^+^/K^+^, H^+^ exchanger family, and only two of them additionally contain the C-terminal cytoplasmic region. Extensive expansion has also been observed for the Pho89 homologs in *H. werneckii*, as we identified six homologs of the Pho89 Na^+^, P_i_ symporter, with each homolog containing at least one PHO4 domain. In contrast with the abundant transporter families mentioned, only four homologs of three *S. cerevisiae* Ena Na^+^ P-type ATPases have been identified in the *H. werneckii* genome (Lenassi et al., 2013). Previously, we identified and characterized two Ena-like P-ATPases (Gorjan and Plemenitaš, 2006). Analysis of the Ena Na^+^ P-type ATPases identified in the genome of *H. werneckii* reveals that each homolog contains all four of the conserved domains found in the *S. cerevisiae* Ena proteins. Considering their multiplication, it appears that Nha transporters are more important than Ena. On the other hand, based on our previous data that demonstrated that *HwENA* genes are highly induced at alkaline pH (Gorjan and Plemenitaš, 2006), we speculate that they have complementary functions: Ena ATPases are more important at high pH, where the Nha antiporters cannot function correctly.

As well as the important role of plasma-membrane transport systems in ion homeostasis, in the cytosol, K^+^ homeostasis and Na^+^ detoxification are also connected to cation transport across the organelle membranes (Arino et al., 2010). In *S. cerevisiae*, endosomal Nhx1 (Nass and Rao, 1999) and Kha1 from the Golgi apparatus (Maresova and Sychrova, 2005) are Na^+^/H^+^ exchangers, similar to Nha1 at the plasma membrane (Prior et al., 1996). The vacuolar Vnx1 (Cagnac et al., 2007) and the mitochondrial Mdm38 and Mrs7 (Nowikovsky et al., 2004; Zotova et al., 2010) have similar Na^+^/K^+^, H^+^ exchanger functions, but different structures.

We found that homologs of Nhx1 and Kha1 are duplicated in the *H. werneckii* genome, all of which contain the domains that are typical for the Na^+^/H^+^ exchanger family. The same has been observed for the Kha1 homologs. We also identified two homologs of transporters with high homology to the Mrs7 and Mdm38 transporters from *S. cerevisiae*. Of the intracellular cation transporters, only the homologs of the vacuolar Vnx1 are enriched in *H. werneckii* in comparison to *S. cerevisiae*. We identified eight homologs of the Vnx1 Na^+^/K^+^, H^+^ antiporter, but the homology of the HwVnx proteins compared to Vnx1 is low (Lenassi et al., 2013).

The activities of many transporters are closely connected to the proton gradients across the membranes, which are generated by the Pma1 P-type ATPase at the plasma membrane (Serrano et al., 1986; Ambesi et al., 2000) and the V-type ATPase at the vacuolar membrane (Graham et al., 2000). Different P-type ATPases use ATP hydrolysis as a source of energy for the transport of ions through the membrane, and they are structurally similar (Kuhlbrandt, 2004).

The enrichment of the transporters responsible for supplying the energy for the cation transporters in *H. werneckii* supports the importance of the complex cation transporter system for combating high environmental Na^+^. We identified four homologs of Pma1 in *H. werneckii*, with each homolog containing three conserved domains that are also found in the *S. cerevisiae* Pma1 and Pma2 proteins. The importance of all four of the *H. werneckii* Pma homologs for cation homeostasis is also supported by expression analysis, as the expression profiles of the *PMA1* and *PMA2* homologs show different levels of response to saline conditions (Lenassi et al., 2013). Comparisons of the expression profiles of the *PMA* genes in *H. werneckii* with those described in *S. cerevisiae* have shown that in *S. cerevisiae*, *PMA1* is not induced by salt stress (Yale and Bohnert, 2001), while in *H. werneckii*, both *PMA1* and *PMA2* have salt-regulated transcription.

The yeast vacuolar ATPase does not only have a crucial role in the acidification of the vacuolar lumen, but it is also important for the correct functioning of other organelles (Arino et al., 2010). In the *H. werneckii* genome, we found homologs of all of the subunits of the *S. cerevisiae* V-ATPase complex. The *H. werneckii* vacuolar subunits in general share a lot of similarity with the *S. cerevisiae* subunits, which is not surprising, as their structures and function have been highly conserved through evolution (Graham et al., 2000). *S. cerevisiae* vacuolar ATPases are localized at different cellular locations; however, it remains to be determined where they are specifically localized in *H. werneckii*. In contrast to the transcription of the *HwPMA*s, which is salt regulated, no such trends have been seen for the expression of the *VMA* homologs under different salinities.

## *Wallemia ichthyophaga* genome analysis

### Characteristics of the genome and the transcriptomes

The genome of *W. ichthyophaga* has been deposited as a Whole Genome Shotgun project at DDBJ/EMBL/GenBank under the accession number APLC00000000 (Zajc et al., 2013). The genome of *W. ichthyophaga* is 9.6 Mb in size, and the sequence currently consists of 101 contigs and 82 scaffolds (Table 1). Most basidiomycetous haploid genomes are more than twice this size (and in some cases, larger by 40-fold or more; Gregory et al., 2007). The closely related species *W. sebi* also has a slightly larger genome (9.8 Mb) (Padamsee et al., 2012). Of the species investigated thus far, only the dandruff- and seborrhoeic-dermatitis-causing *Malassezia globosa* has a smaller genome (9.0 Mb; Gregory et al., 2007). The compactness of the genome of *W. ichthyophaga* is reflected in its low level of repetitive sequences (1.67%), and high density of genes (514 genes/Mb scaffold) (Zajc et al., 2013). This is only slightly lower than *W. sebi* (538 genes/Mb), but more than in *M. globosa* (476 genes/Mb). This means that the coding DNA sequences in *W. ichthyophaga* cover almost three quarters of the genome. The GC content in *W. ichthyophaga* is 45.35%, while in *W. sebi* this is even lower, at 40.01% (Table 1). The absolute number of predicted proteins in *W. ichthyophaga* (4884; Zajc et al., 2013) is also unusually small for a basidiomycete (where more than 10,000 proteins are not uncommon), and is in the range observed for *Escherichia coli* (Lukjancenko et al., 2010). For comparison, the *W. sebi* genome codes for 5284 proteins, while *M. globosa* contains 4285 proteins. Interestingly, the reduction in genome size and gene number is not accompanied by a reduction in intron number, such as has been reported for some other fungi with small genomes (Kelkar and Ochman, 2012).

It has not been possible to assign functions for an unproportionally large number of the proteins that are found in *W. ichthyophaga* but not in *W. sebi*. With searches through the Pfam database, three quarters of these proteins could not be classified into any of the protein families (Zajc et al., 2013). Among those that could be identified, there were several proteins related to DNA processing and DNA damage.

The sequencing of the transcriptomes of *W. ichthyophaga* grown in 10% and 30% (w/v) NaCl has revealed that 13.1% of the genes are differentially expressed under these conditions (Zajc et al., 2013). Of these, two thirds are more expressed at lower salinity. Alternative splicing, which has been identified as intron retention, was detected for 15.0% of the genes, and in more than half of the cases (51.6%), alternative splicing was detected only at one of the two tested salinities (Zajc et al., 2013).

### Compatible solute management

The strategy of osmo-adaptation of both the extremely halotolerant *H. werneckii* and the halophilic *W. ichthyophaga* is the accumulation of a mixture of polyols that act as compatible solutes (Figure 1). The main osmotically regulated polyol of both *H. werneckii* and *W. ichthyophaga* is glycerol, the levels of which are increased with increasing salinity and decreased after hypo-osmotic shock (Kogej et al., 2007; Zajc et al., 2014). In addition to glycerol, we have reported erythritol, arabitol, and mannitol in *H. werneckii* (Plemenitaš et al., 2008), and smaller amounts of arabitol, and traces of mannitol in *W. ichthyophaga* (Zajc et al., 2014). The genes for the enzymes known to be involved in compatible solute management are found in the genome of *W. ichthyophaga* (Table 2). These are present in several copies, with the exception of the glycerol-3-phosphatase Gpp. *W. ichthyophaga* contains a homolog of *GPD1*, *WiGPD1*, the expression of which is salt-induced (Lenassi et al., 2011). A second homolog was also found by searching the genome. When compared with the homologs of *H. werneckii*, the expression level of *WiGPD1* is lower, and the response to hyperosmotic shock is slower (Lenassi et al., 2011). Expression of *WiGPD1* in *S. cerevisiae* boosted the osmotolerance of the *gpd1* and *gpd1gpd2* mutants. As was reported for homologs from *H. werneckii*, *WiGPD1* lacks the N-terminal peroxisomal targeting (PTS2) sequence (Lenassi et al., 2011), which is important for peroxisome localization of WiGpd1 (Jung et al., 2010). This might mean that WiGpd1 remains in the cytosol, which would be an advantage when living in extremely saline environments, as it is this fraction that is important for the synthesis of the compatible solutes (Lenassi et al., 2011). During hyperosmotic shock, *S. cerevisiae* counteracts glycerol leakage by its active re-import using the protein Stl1, a glycerol/H^+^ symporter in the plasma membrane (Ferreira et al., 2005). Similarly, the aquaglyceroporin channel Fps1 remains closed (while it opens during hypo-osmotic shock, to facilitate expulsion of excess glycerol) (Luyten et al., 1995). In *W. ichthyophaga*, four homologs of Stl1 have been found (Figure 1, Table 2), as well as three aquaglyceroporin-related proteins.

In the basidiomycete *Agaricus bisporus*, the solute D-mannitol is synthesized from fructose via a reduction step that is catalysed by two NADP-dependent mannitol dehydrogenases (Stoop and Mooibroek, 1998). *W. ichthyophaga* also contains two homologs of D-arabinitol-2-dehydrogenases, which are used in other fungi for the production of arabitol from an intermediate of the pentose phosphate pathway, D-ribulose-5-phosphate.

### Transport of alkali metal ions

In line with the strategy of compatible solutes, the intracellular levels of K^+^ and Na^+^ in *W. ichthyophaga* remain low at constant salinities (not above 30 nmol/mg dry biomass) (Zajc et al., 2013), even when compared to *H. werneckii* (not above 180 nmol/mg dry biomass) (Kogej et al., 2007). However, when under hyperosmotic shock, the levels of both cations increase significantly in *W. ichthyophaga*, indicating its poor capability to adjust to changing environments. The ratio between these cations decreases with increasing salinity, due to the rising levels of Na^+^ and the lowering of K^+^. However, the intracellular K^+^/Na^+^ ratio is higher across the whole salinity range in *W. ichthyophaga* compared to *H. werneckii* (Figure 1) and some other halotolerant fungi (e.g., *Aureobasidium pullulans*, *Debaryomyces hansenii*). In addition, the K^+^/Na^+^ decrease over the salinity range is less steep in *W. ichthyophaga* compared to *H. werneckii*. The growth performance of *W. ichthyophaga* is greatest when the Na^+^ content exceeds that of K^+^ (Zajc et al., 2014). This indicates that these intracellular concentrations of Na^+^ ions are not toxic to the cells.

Data from the genome show that there are only a low number of cation transporters, except for the enriched protein family of P-type ATPases (Figure 1, Table 2). Also, expression of the cation transporters is low and independent of salt, with only three minor exceptions (described below). This is probably associated with the life of *W. ichthyophaga* at relatively constant (although extremely high) salinities. Nevertheless, in its genome we observed a significant enrichment of the cation-transporting ATPases family. The identified proteins of this family are three H^+^ and two Na^+^ P-type ATPases (all of which are assumed to be located at the plasma membrane), two Ca^2+^ P-type ATPases (vacuolar Pmc1 and Pmr1 from the Golgi apparatus), and a putative transporter of unknown specificity (Zajc et al., 2013). The *W. ichthyophaga* genome encodes three putative Pma proton pumps, while *W. sebi* contains only two (Zajc et al., 2013).

In environments with high concentrations of Na^+^ salts, the cell must prevent the intracellular accumulation of the highly toxic Na^+^, without lowering the levels of K^+^. This is achieved by a variety of other secondary active transporters. *W. ichthyophaga* contains homologs of most known transporters from *S. cerevisiae* (Arino et al., 2010) and unconventional yeast (Ramos et al., 2011), as either those located on intracellular membranes (Kha1, Mrs7/Mdm37, Nhx1, Pmc1, Pmr1, Vnx1, Vma1) or at the plasma membrane (Ena, Nha1, Pho89, Pma, Trk1).

Judging by the genes that encode alkali metal cation transporters, the extremely halotolerant ascomycete *H. werneckii* and *W. ichthyophaga* use different salt-combating strategies. As described above, in *H. werneckii*, the numbers of most of the plasma-membrane alkali cation transporters are substantially increased. In *W. ichthyophaga* this is not the case (Figure 1, Table 2). *W. ichthyophaga* contains only one Trk homolog (inward K^+^ transporter, with eight copies in *H. werneckii*) and no Tok homologs (outward K^+^ channel, with four copies in *H. werneckii*). Similarly, *W. ichthyophaga* has only two Nha homologs (Na^+^/K^+^ proton antiporters) and one Pho89 (Na^+^/P_i_ symporter), while *H. werneckii* contains eight and six, respectively (Lenassi et al., 2013; Zajc et al., 2013).

Active import of K^+^ might contribute to ion homeostasis in hypersaline environments, and this would complement the action of passive K^+^ channels. The known active transporters are K^+^-H^+^symporters (Hak symporters) and K^+^(Na^+^)-ATPase (Acu, alkali cation uptake transporters) (Benito et al., 2004; Ramos et al., 2011). While *H. werneckii* has no homologs of either of these transporter types (Lenassi et al., 2013), *W. ichthyophaga* contains two possible homologs of the otherwise rare Acu ATPases, of which only one contains a P-type ATPase domain (Zajc et al., 2013).

Several different membrane alkali metal transporters (mainly cation/H^+^ antiporters) are also located on the organelle membranes: the Golgi apparatus (Kha1), mitochondria (Mdm38 or Mrs7), endosomes (Nhx1), and vacuole (Vnx1) (Arino et al., 2010). In *W. ichthyophaga*, there are single-copy genes of all of these proteins (with the exception of the duplicated Kha1).

*W. ichthyophaga* can live at extremely high salinity; however, the above-described findings indicate that the salinity remains relatively constant and thus no rapid responses are needed. Under constant conditions, there is no need for a quick release of surplus K^+^, and thus the absence of the Tok outward K^+^ channel might not be detrimental. On the other hand, *W. ichthyophaga* contains three homologs of aquaglyceroporins (Zajc et al., 2013), which fulfil the need for rapid expulsion of the accumulated compatible solute glycerol (Luyten et al., 1995). Two explanations are possible: (1) *W. ichthyophaga* might deal with hypo-osmotic shock-related K^+^ expulsion in other ways than in other fungi; and (2) the aquaglyceroporin channels might serve some other functions than expulsion of glycerol during the shock.

In cells grown at 10 and 30% (w/v) NaCl, the large majority of the genes that encode metal-cation transporters are not differentially expressed. This is not as expected, since these transporters are believed to have crucial roles in adaptation to salt. The only exceptions are a homolog of the Pho89 Na^+^/P_i_ symporter, which shows elevated expression at high salinity, and a putative P-type Na^+^ ATPase and a possible Acu K^+^ importer, with the expression of both of these latter higher at low salinity. This is in stark contrast, for example, with other halotolerant fungi (e.g., *D. hansenii*, *H. werneckii*), where even at different constant salinities, differential expression of P-type H^+^ and Na^+^ ATPases has been observed (Almagro et al., 2001; Gorjan and Plemenitaš, 2006; Lenassi et al., 2013). Furthermore, in *W. ichthyophaga* the expression of transporter coding genes is relatively low: of the total of 4884 genes, neither the Na^+^-exporting P-type ATPases nor the two Na^+^/H^+^ antiporters are among the 2000 most-expressed genes at high salinity. The possible post-transcriptional control of all of these genes remains to be investigated.

Continuous removal and/or compartmentalization of Na^+^ at a constant high salinity is extremely demanding energetically. The low numbers of transporters in *W. ichthyophaga* might reflect the relatively low adaptive potential of this species to changes in salt concentrations and/or its specialization with other mechanisms that are energetically more efficient. The apparent transcriptional non-responsiveness of transporters to salt can lead to similar conclusions. This would mean that the halophilic strategy of *W. ichthyophaga*, which is a unique example of a narrowly specialized fungal halophile (Gostinčar et al., 2010), is substantially different from that of *H. werneckii*, which contains a collection of K^+^ channels and can adapt to a wide salinity range (Zajc et al., 2013).

### Hydrophobins

The analysis of the *W. ichthyophaga* genome has revealed a significant expansion of seven protein families and contraction of 19. The most interesting of the expanded families are the hydrophobins, which are proteins that potentially have a role in the particular morphological adaptations of *W. ichthyophaga* (Figure 1; Zajc et al., 2013).

*W. ichthyophaga* has a characteristic morphology that can be seen in many stress-tolerant species: compact multicellular clumps that are similar to sarcinae (Zalar et al., 2005b). This morphology has been observed in, and it is believed to enhance survival in, high-stress environments (Wollenzien et al., 1995; Palkova and Vachova, 2006; Gostinčar et al., 2011). These cells have an abundant cover of extracellular polysaccharides (Kralj Kunčič et al., 2010) that can serve as protectants during desiccation (Selbmann et al., 2005), and possibly also when exposed to high concentrations of salt (Zajc et al., 2013). Additionally, at high salinity the morphology of *W. ichthyophaga* cells changes strikingly. The size of the meristematic cell clumps increases four-fold, and the cell walls become three-fold thicker, which also substantially decreases the intracellular volume (Kralj Kunčič et al., 2010).

The hydrophobins are proteins in the cell wall of filamentous fungi. These small (≤20 kDa) and amphipathic molecules (Linder et al., 2005) are involved in a range of processes of cellular growth and development (Wosten, 2001). Possibly as a reflection of the many different roles they have, the hydrophobin genes are often present in multiple different copies. From an estimated 15 hydrophobin genes in the last common ancestor of *W. ichthyophaga* and *W. sebi*, these genes are enriched to 26 in *W. ichthyophaga*, while the number has fallen to 12 in *W. sebi*. This is the most significant protein family expansion in the genome of *W. ichthopyhaga* (Zajc et al., 2013).

The hydrophobins that have been identified in *W. ichthyophaga* and *W. sebi* contain the characteristic hydrophobin pattern of the conserved spacing of eight cysteine residues (Zajc et al., 2013) that form four disulphide bridges (Hektor and Scholtmeijer, 2005; Linder et al., 2005). These hydrophobins in both *W. ichthyophaga* and *W. sebi* contain a high proportion of acidic amino acids compared to homologs from other fungi (Zajc et al., 2013). This is similar to the archaeal halophilic proteins (Madern et al., 2000) and it might represent an adaptation to salt exposure. If acidic amino acids are exposed on a protein surface, they can bind salt and water, and thus help to avoid salt-induced changes in conformation that would lead to loss of activity (Siglioccolo et al., 2011). This might also be the case for the hydrophobins of *W. ichthyophaga*, as these are among the few of its proteins that are actually exposed to high concentrations of NaCl and are not protected in the intracellular compatible-solute-rich environment. Interestingly, half of the genes encoding hydrophobins are differentially expressed during growth of *W. ichthyophaga* at different salinities, although their responses are not the same: at high salinity some of these hydrophobins have higher expression, and some have lower expression (Zajc et al., 2013).

The hydrophobins can self-assemble into amphipathic monolayers on hydrophobic–hydrophilic interfaces, which is crucial for their diverse functions. With their help, the cell can attach to hydrophobic surfaces, break through a water-air interface, and avoid water-logging without impeding the exchange of gasses. Hydrophobins can also strengthen the cell wall and make it more rigid, and they also impact on the movement of solutes (Wosten, 2001; Bayry et al., 2012). It is not difficult to imagine that these functions will be beneficial to cells exposed to high salinity (Zajc et al., 2013). Under these conditions, the leakage of compatible solutes and intrusion of toxic salt ions are among the greatest challenges to the cell. Increased strength and rigidity of the cell wall will help the cell to survive the structural stress that can be imposed by changes in the environmental osmolarity.

The hydrophobins might also have a role in the characteristic sarcina-like morphology of *W. ichthyophaga*, which is similar to *Fusarium verticillioides*, where the hydrophobins trigger microconidial chain formation (Fuchs et al., 2004). This might additionally be involved in the aggregation of *W. ichthyophaga* cells into compact cell clusters, as is characteristic of *W. ichthyophaga*, and which is probably a form of salt-stress response (Gostinčar et al., 2010). This characteristic sarcina-like morphology is considered to be among the main haloadaptations of *W. ichthyophaga*. Indeed, both the changes in the cell wall and the formation of multicellular structures have previously been suggested to be among the main adaptations of *W. ichthyophaga* to hypersaline environments (Kralj Kunčič et al., 2010).

### Genomic evidence of asexuality

Traditional mycological approaches have not lead to any descriptions of mating behaviors for either *H. werenckii* or the *Wallemia* spp.. In both cases, no descriptions of fruiting bodies or reports of teleomorphs can be found in the literature. Basidomycetous fungi have a tetrapolar *MAT* locus. Two additional unlinked loci encode the homeodomain-containing transcription factors and pheromone/pheromone receptors. In some cases, other mating architectures have evolved through the expansion and fusion of these *MAT* loci (Lee et al., 2010). The genome of *W. sebi* contains a single mating-type locus and lacks only a few meiosis-specific genes, which suggested that it can undergo sexual reproduction (Padamsee et al., 2012). This is not the case for *W. ichthyophaga* (Zajc et al., 2013). Searches of the *W. ichthyophaga* genome for proteins that are similar to the gene products involved in mating in other basidiomycetes has resulted in the identification of only a few proteins, and even these are very dissimilar to proteins from other fungi. No discernible mating locus has been identified, and only three of eight meiosis-specific genes (as listed in Malik et al., 2008) have been found (Zajc et al., 2013).

Asexuality in fungi is not unusual. Asexual reproduction saves energy, as it eliminates the need to produce gametes and attractants, and this might be useful in extreme environments where careful management of energy is of key importance. In habitats that do not undergo major changes over longer time scales, this would also prevent the drowning of specific adaptations of local populations in the larger gene pool of the species (Gostinčar et al., 2010; Sun and Heitman, 2011). Therefore, in the case of these extremophilic fungal species, an asexual lifestyle might have evolutionary advantages (Gostinčar et al., 2010). This appears to be the case for *W. ichthyophaga*, and this might also be true for *H. werneckii*. While a putative mating type locus has been found in the genome of *H. werneckii*, no sexual stage of this species has been described to date. As mating genes can have roles outside of mating (Srikantha et al., 2012), their presence in itself is not confirmation of sexual reproduction in a species. The potential role of sexual reproduction in *H. werneckii* therefore needs to be further investigated.

## Conclusions

The extremely halotolerant and adaptable *H. werneckii* and the obligately halophilic *W. ichthyophaga* live in environments that are defined by low a_w_ and high concentrations of toxic inorganic ions. To withstand these harsh environmental conditions they use some common molecular mechanisms and also some specific molecular mechanisms. A model of the key adaptations in *H. werneckii* and *W. ichthyophaga* that have been discussed here is illustrated in Figure 1.

When a cell is exposed to a high osmolarity (salinity) environment, it has to react rapidly to the consequent loss of water. In *H. werneckii* and *W. ichthyophaga* this is achieved by the synthesis of glycerol and a few other compatible solutes (Figure 1). The expression of the key enzyme in glycerol synthesis, Gpd1, is under the control of the HOG signaling pathway, which is also important for other aspects of adaptation to high osmolarity environments. The key proteins of the HOG pathway are conserved in *H. werneckii* and *W. ichthyophaga*, although the HOG pathway architecture and regulation is different. While all of the HOG pathway components are present in at least two isoforms in *H. werneckii*, there are only two isoforms of the Hog1 kinase in *W. ichthyophaga* (Figure 1). In *H. werneckii*, HwHog1 is only phosphorylated under extracellular conditions of ≥3 M NaCl (Turk and Plemenitaš, 2002), while in *W. ichthyophaga*, the WiHog1 kinase is constitutively phosphorylated under optimal osmotic conditions and is dephosphorylated upon hyperosmotic or hypo-osmotic shock (Konte and Plemenitaš, 2013). In *H. werneckii*, HwHog1 promotes differential induction or repression of osmoresponsive genes, depending on the osmolarity, and also by physically interacting with chromatin and RNA polymerase II (Vaupotič and Plemenitaš, 2007). While this specific architecture of the HOG pathway might represent the background for the extreme halotolerance of *H. werneckii*, the constitutive phosphorylation of the Hog1-like kinase in *W. ichthyophaga* might support its obligate halophilic nature.

In many natural hypersaline environments, the concentrations of toxic Na^+^ ions are far greater than those of K^+^ ions, and therefore the mechanisms that maintain the stable and high intracellular K^+^/Na^+^ ratio are crucial for the survival in such environments. Both, *H. werneckii* and *W. ichthyophaga* can maintain high K^+^/Na^+^ ratios over a wide range of environmental Na^+^ concentrations. In *H. werneckii*, this homeostasis is maintained by regulated transport of K^+^ and Na^+^ across the plasma membrane, as cation transporters are diverse and highly enriched in this fungus (Figure 1). *W. ichthyophaga* also regulates the entry and expulsion of cations; however, it mainly prevents their entry by dynamic cell-wall restructuring. An explanation for these observed differences in the way that these fungi combat the toxic environmental Na^+^ might be the need for *H. werneckii* to adapt rapidly to highly dynamic concentrations of NaCl (and other salts) that it typically encounters in its natural environment, while *W. ichthyophaga* thrives instead under continuously high salinity.

Large genetic redundancy is an important characteristic of *H. werneckii*, which has presumably resulted from the whole genome duplication. Although the whole genome duplication that is seen in *H. werneckii* is not uncommon for fungi, it is interesting that this duplication has not yet been followed by selective gene loss, as the large majority of genes in *H. werneckii* is still present in two copies. Such redundancy might be an excellent reservoir for cryptic genetic variability, which is of importance under stress environments that require good adaptability (Gostinčar et al., 2010). This might be especially the case, as the extent of sexual recombination as a means of generating genetic diversity in *H. werneckii* is still not known. Indeed, in *H. werneckii* there is a recognizable putative mating type locus, which indicates the possibility of (although so far not observed) a sexual reproduction cycle, while *W. ichthyophaga* lacks the cellular machinery for sexual reproduction altogether (Figure 1).

We believe that the recently published genome and transcriptome sequences of *H. werneckii* and *W. ichthyophaga* will accelerate research into the osmotic strategies of these fungi that can thrive in high salinity environments that allow the survival of only the most specialized minority of eukaryotes and prokaryotes.

### Conflict of interest statement

The authors declare that the research was conducted in the absence of any commercial or financial relationships that could be construed as a potential conflict of interest.

## References

[B1] Almagro A. Prista C. Benito B. Loureiro-Dias M. C. Ramos J. (2001). Cloning and expression of two genes coding for sodium pumps in the salt-tolerant yeast *Debaryomyces hansenii*. J. Bacteriol. 183, 3251–3255 10.1128/JB.183.10.3251-3255.2001 11325955 PMC95227

[B2] Ambesi A. Miranda M. Petrov V. V. Slayman C. W. (2000). Biogenesis and function of the yeast plasma-membrane H^+^-ATPase. J. Exp. Biol. 203(pt 1), 155–160 10600684 10.1242/jeb.203.1.155

[B3] Arino J. Ramos J. Sychrova H. (2010). Alkali metal cation transport and homeostasis in yeasts. Microbiol. Mol. Biol. Rev. 74, 95–120 10.1128/MMBR.00042-09 20197501 PMC2832347

[B4] Bahn Y. S. Geunes-Boyer S. Heitman J. (2007). Ssk2 mitogen-activated protein kinase kinase kinase governs divergent patterns of the stress-activated Hog1 signaling pathway in *Cryptococcus neoformans*. Eukaryot. Cell 6, 2278–2289 10.1128/EC.00349-07 17951522 PMC2168243

[B5] Bayry J. Aimanianda V. Guijarro J. I. Sunde M. Latge J. P. (2012). Hydrophobins-unique fungal proteins. PLoS Pathog. 8:e1002700 10.1371/journal.ppat.1002700 22693445 PMC3364958

[B6] Benito B. Garciadeblas B. Schreier P. Rodriguez-Navarro A. (2004). Novel P-type ATPases mediate high-affinity potassium or sodium uptake in fungi. Eukaryot. Cell 3, 359–368 10.1128/EC.3.2.359-368.2004 15075266 PMC387655

[B7] Blomberg A. Adler L. (1992). Physiology of osmotolerance in fungi. Adv. Microb. Physiol. 33, 145–212 10.1016/S0065-2911(08)60217-9 1636508

[B8] Brewster J. L. de Valoir T. Dwyer N. D. Winter E. Gustin M. C. (1993). An osmosensing signal transduction pathway in yeast. Science 259, 1760–1763 10.1126/science.7681220 7681220

[B9] Cagnac O. Leterrier M. Yeager M. Blumwald E. (2007). Identification and characterization of Vnx1p, a novel type of vacuolar monovalent Cation/H^+^ antiporter of *Saccharomyces cerevisiae*. J. Biol. Chem. 282, 24284–24293 10.1074/jbc.M703116200 17588950

[B10] Cantrell S. A. Casillas-Martinez L. Molina M. (2006). Characterization of fungi from hypersaline environments of solar salterns using morphological and molecular techniques. Mycol. Res. 110, 962–970 10.1016/j.mycres.2006.06.005 16904880

[B11] de Hoog G. S. Gerrits van den Ende A. H. (1992). Nutritional pattern and eco-physiology of *Hortaea werneckii*, agent of human tinea nigra. Anton. Leeuw. 62, 321–329 10.1007/BF00572601 1283680

[B12] de Hoog G. S. Guého E. (1998). Agents of white piedra, black piedra and tinea nigra, in Topley and Wilsons Microbiology and Microbial Infections, Vol. 4, eds Ajello L. Hay R. J. (London, UK: Arnold Publications), 191–197

[B13] Ferreira C. van Voorst F. Martins A. Neves L. Oliveira R. Kielland-Brandt M. C. (2005). A member of the sugar transporter family, Stl1p is the glycerol/H^+^ symporter in *Saccharomyces cerevisiae*. Mol. Biol. Cell 16, 2068–2076 10.1091/mbc.E04-10-0884 15703210 PMC1073684

[B14] Fettich M. Lenassi M. Veranič P. Gunde-Cimerman N. Plemenitaš A. (2011). Identification and characterization of putative osmosensors, HwSho1A and HwSho1B, from the extremely halotolerant black yeast *Hortaea werneckii*. Fungal Genet. Biol. 48, 475–484 10.1016/j.fgb.2011.01.011 21281727

[B15] Fuchs U. Czymmek K. J. Sweigard J. A. (2004). Five hydrophobin genes in *Fusarium verticillioides* include two required for microconidial chain formation. Fungal Genet. Biol. 41, 852–864 10.1016/j.fgb.2004.04.004 15288021

[B16] Gorjan A. Plemenitaš A. (2006). Identification and characterization of ENA ATPases HwENA1 and HwENA2 from the halophilic black yeast *Hortaea werneckii*. FEMS Microbiol. Lett. 265, 41–50 10.1111/j.1574-6968.2006.00473.x 17034413

[B17] Gostinčar C. Grube M. de Hoog S. Zalar P. Gunde-Cimerman N. (2010). Extremotolerance in fungi: evolution on the edge. FEMS Microbiol. Ecol. 71, 2–11 10.1111/j.1574-6941.2009.00794.x 19878320

[B18] Gostinčar C. Lenassi M. Gunde-Cimerman N. Plemenitaš A. (2011). Fungal adaptation to extremely high salt concentrations. Adv. Appl. Microbiol. 77, 71–96 10.1016/B978-0-12-387044-5.00003-0 22050822

[B19] Graham L. A. Powell B. Stevens T. H. (2000). Composition and assembly of the yeast vacuolar H^+^-ATPase complex. J. Exp. Biol. 203, 61–70 10600674 10.1242/jeb.203.1.61

[B20] Gregory T. R. Nicol J. A. Tamm H. Kullman B. Kullman K. Leitch I. J. (2007). Eukaryotic genome size databases. Nucleic Acids Res. 35, 332–338 10.1093/nar/gkl828 17090588 PMC1669731

[B21] Gunde-Cimerman N. Plemenitaš A. (2006). Ecology and molecular adaptations of the halophilic black yeast *Hortaea werneckii*. Rev. Environ. Sci. Biotechnol. 5, 323–331 10.1007/s11157-006-9105-0

[B22] Gunde-Cimerman N. Zalar P. de Hoog S. Plemenitaš A. (2000). Hypersaline waters in salterns - natural ecological niches for halophilic black yeasts. FEMS Microbiol. Ecol. 32, 235–240 10.1016/S0168-6496(00)00032-5 10858582

[B23] Hektor H. J. Scholtmeijer K. (2005). Hydrophobins: proteins with potential. Curr. Opin. Biotechnol. 16, 434–439 10.1016/j.copbio.2005.05.004 15950452

[B24] Hibbett D. S. Binder M. Bischoff J. F. Blackwell M. Cannon P. F. Eriksson O. E. (2007). A higher-level phylogenetic classification of the Fungi. Mycol. Res. 111, 509–547 10.1016/j.mycres.2007.03.004 17572334

[B25] Hohmann S. Krantz M. Nordlander B. (2007). Yeast osmoregulation. Method Enzymol. 428, 29–45 10.1016/S0076-6879(07)28002-4 17875410

[B26] Iwatsu T. U. Udagawa S. (1988). *Hortaea werneckii* isolated from sea-water. Jpn. J. Med. Mycol. 29, 142–145 10.3314/jjmm1960.29.142

[B27] Jung S. Marelli M. Rachubinski R. A. Goodlett D. R. Aitchison J. D. (2010). Dynamic changes in the subcellular distribution of Gpd1p in response to cell stress. J. Biol. Chem. 285, 6739–6749 10.1074/jbc.M109.058552 20026609 PMC2825468

[B28] Kelkar Y. D. Ochman H. (2012). Causes and consequences of genome expansion in fungi. Genome Biol. Evol. 4, 13–23 10.1093/gbe/evr124 22117086 PMC3267396

[B29] Kogej T. Ramos J. Plemenitaš A. Gunde-Cimerman N. (2005). The halophilic fungus *Hortaea werneckii* and the halotolerant fungus *Aureobasidium pullulans* maintain low intracellular cation concentrations in hypersaline environments. Appl. Environ. Microbiol. 71, 6600–6605 10.1128/AEM.71.11.6600-6605.2005 16269687 PMC1287720

[B30] Kogej T. Stein M. Volkmann M. Gorbushina A. A. Galinski E. A. Gunde-Cimerman N. (2007). Osmotic adaptation of the halophilic fungus *Hortaea werneckii*: role of osmolytes and melanization. Microbiology 153, 4261–4273 10.1099/mic.0.2007/010751-0 18048939

[B31] Konte T. Plemenitaš A. (2013). The HOG signal transduction pathway in the halophilic fungus *Wallemia ichthyophaga*: identification and characterisation of MAP kinases WiHog1A and WiHog1B. Extremophiles 17, 623–636 10.1007/s00792-013-0546-4 23712906

[B32] Kralj Kunčič M. Kogej T. Drobne D. Gunde-Cimerman N. (2010). Morphological response of the halophilic fungal genus *Wallemia* to high salinity. Appl. Environ. Microbiol. 76, 329–337 10.1128/AEM.02318-09 19897760 PMC2798636

[B33] Kralj Kunčič M. Zajc J. Drobne D. Pipan Tkalec Z. Gunde-Cimerman N. (2013). Morphological responses to high sugar concentrations differ from adaptation to high salt concentrations in the xerophilic fungi *Wallemia spp*. Fungal Biol. 117, 466–478 10.1016/j.funbio.2013.04.003 23931114

[B34] Kuhlbrandt W. (2004). Biology, structure and mechanism of P-type ATPases. Nat. Rev. Mol. Cell Biol. 5, 282–295 10.1038/nrm1354 15071553

[B35] Lee S. C. Ni M. Li W. J. Shertz C. Heitman J. (2010). The evolution of sex: a perspective from the fungal kingdom. Microbiol. Mol. Biol. R 74, 298–340 10.1128/MMBR.00005-10 20508251 PMC2884414

[B36] Lenassi M. Gostinčar C. Jackman S. Turk M. Sadowski I. Nislow C. (2013). Whole genome duplication and enrichment of cation transporters are associated with the halotolerant lifestyle of the black yeast *Hortaea werneckii*. PLoS ONE 30:e71328 10.1371/journal.pone.0071328 23977017 PMC3744574

[B37] Lenassi M. Plemenitas A. (2007). Novel group VII histidine kinase HwHhk7B from the halophilic fungi *Hortaea werneckii* has a putative role in osmosensing. Curr. Genet. 51, 393–405 10.1007/s00294-007-0131-4 17435999

[B38] Lenassi M. Vaupotič T. Gunde-Cimerman N. Plemenitaš A. (2007). The MAP kinase HwHog1 from the halophilic black yeast *Hortaea werneckii*: coping with stresses in solar salterns. Saline Systems 3, 3 10.1186/1746-1448-3-3 17349032 PMC1828057

[B39] Lenassi M. Zajc J. Gostinčar C. Gorjan A. Gunde-Cimerman N. Plemenitaš A. (2011). Adaptation of the glycerol-3-phosphate dehydrogenase Gpd1 to high salinities in the extremely halotolerant *Hortaea werneckii* and halophilic *Wallemia ichthyophaga*. Fungal Biol. 115, 959–970 10.1016/j.funbio.2011.04.001 21944208

[B40] Linder M. B. Szilvay G. R. Nakari-Setala T. Penttila M. E. (2005). Hydrophobins: the protein-amphiphiles of filamentous fungi. FEMS Microbiol. Rev. 29, 877–896 10.1016/j.femsre.2005.01.004 16219510

[B41] Lukjancenko O. Wassenaar T. M. Ussery D. W. (2010). Comparison of 61 sequenced *Escherichia coli* genomes. Microb. Ecol. 60, 708–720 10.1007/s00248-010-9717-3 20623278 PMC2974192

[B42] Luyten K. Albertyn J. Skibbe W. F. Prior B. A. Ramos J. Thevelein J. M. (1995). Fps1, a yeast member of the mip family of channel proteins, is a facilitator for glycerol uptake and efflux and is inactive under osmotic-stress. EMBO J. 14, 1360–1371 7729414 10.1002/j.1460-2075.1995.tb07122.x PMC398221

[B43] Madern D. Ebel C. Zaccai G. (2000). Halophilic adaptation of enzymes. Extremophiles 4, 91–98 10.1007/s007920050142 10805563

[B44] Maeda T. Wurglermurphy S. M. Saito H. (1994). A 2-component system that regulates an osmosensing Map kinase cascade in yeast. Nature 369, 242–245 10.1038/369242a0 8183345

[B45] Malik S. B. Pightling A. W. Stefaniak L. M. Schurko A. M. Logsdon J. M. (2008). An expanded inventory of conserved meiotic genes provides evidence for sex in *Trichomonas vaginalis*. PLoS ONE 3:e2879 10.1371/journal.pone.0002879 18663385 PMC2488364

[B46] Maresova L. Sychrova H. (2005). Physiological characterization of *Saccharomyces cerevisiae kha1* deletion mutants. Mol. Microbiol. 55, 588–600 10.1111/j.1365-2958.2004.04410.x 15659172

[B47] Matheny P. B. Gossmann J. A. Zalar P. Kumar T. K. A. Hibbett D. S. (2006). Resolving the phylogenetic position of the Wallemiomycetes: an enigmatic major lineage of Basidiomycota. Can. J. Bot. 84, 1794–1805 10.1139/b06-128

[B48] Meena N. Kaur H. Mondal A. K. (2010). Interactions among HAMP domain repeats act as an osmosensing molecular switch in group III hybrid histidine kinases from Fungi. J. Biol. Chem. 285, 12121–12132 10.1074/jbc.M109.075721 20164185 PMC2852951

[B49] Mok W. Y. Castelo F. P. Barreto Da Silva M. S. (1981). Occurrence of *Exophiala werneckii* on salted freshwater fish *Osteoglossum bicirrhosum*. Int J Food Sci Technol 16, 505–512 10.1111/j.1365-2621.1981.tb01843.x

[B50] Nass R. Rao R. (1999). The yeast endosomal Na^+^/H^+^ exchanger, Nhx1, confers osmotolerance following acute hypertonic shock. Microbiology 145, 3221–3228 10589731 10.1099/00221287-145-11-3221

[B51] Nowikovsky K. Froschauer E. M. Zsurka G. Samaj J. Reipert S. Kolisek M. (2004). The LETM1/YOL027 gene family encodes a factor of the mitochondrial K^+^ homeostasis with a potential role in the Wolf-Hirschhorn syndrome. J. Biol. Chem. 279, 30307–30315 10.1074/jbc.M403607200 15138253

[B52] Ohm R. A. Feau N. Henrissat B. Schoch C. L. Horwitz B. A. Barry K. W. (2012). Diverse lifestyles and strategies of plant pathogenesis encoded in the genomes of eighteen Dothideomycetes fungi. PLoS Pathog. 8:e1003037 10.1371/journal.ppat.1003037 23236275 PMC3516569

[B53] Padamsee M. Kumar T. K. Riley R. Binder M. Boyd A. Calvo A. M. (2012). The genome of the xerotolerant mold *Wallemia sebi* reveals adaptations to osmotic stress and suggests cryptic sexual reproduction. Fungal Genet. Biol. 49, 217–226 10.1016/j.fgb.2012.01.007 22326418

[B54] Palkova Z. Vachova L. (2006). Life within a community: benefit to yeast long-term survival. FEMS Microbiol. Rev. 30, 806–824 10.1111/j.1574-6976.2006.00034.x 16911045

[B55] Petelenz-Kurdziel E. Eriksson E. Smedh M. Beck C. Hohmann S. Goksor M. (2011). Quantification of cell volume changes upon hyperosmotic stress in *Saccharomyces cerevisiae*. Integr. Biol. 3, 1120–1126 10.1039/c1ib00027f 22012314

[B56] Petrovič U. Gunde-Cimerman N. Plemenitaš A. (2002). Cellular responses to environmental salinity in the halophilic black yeast *Hortaea werneckii*. Mol. Microbiol. 45, 665–672 10.1046/j.1365-2958.2002.03021.x 12139614

[B57] Plemenitaš A. Vaupotič T. Lenassi M. Kogej T. Gunde-Cimerman N. (2008). Adaptation of extremely halotolerant black yeast *Hortaea werneckii* to increased osmolarity: a molecular perspective at a glance. Stud. Mycol. 61, 67–75 10.3114/sim.2008.61.06 19287528 PMC2610308

[B58] Prior C. Potier S. Souciet J. L. Sychrova H. (1996). Characterization of the NHA1 gene encoding a Na^+^/H^+^-antiporter of the yeast *Saccharomyces cerevisiae*. FEBS Lett. 387, 89–93 10.1016/0014-5793(96)00470-X 8654575

[B59] Ramos J. Arino J. Sychrova H. (2011). Alkali-metal-cation influx and efflux systems in nonconventional yeast species. FEMS Microbiol. Lett. 317, 1–8 10.1111/j.1574-6968.2011.02214.x 21241357

[B60] Saito H. Posas F. (2012). Response to hyperosmotic stress. Genetics 192, 289–318 10.1534/genetics.112.140863 23028184 PMC3454867

[B61] Samson R. A. Hoekstra E. S. Frisvad J. C. (2004). Introduction to Food- and Airborne Fungi. Utrecht: Centraalbureau voor Schimmelcultures

[B62] Selbmann L. de Hoog G. S. Mazzaglia A. Friedmann E. I. Onofri S. (2005). Fungi at the edge of life: cryptoendolithic black fungi from Antarctic desert. Stud. Mycol. 1–32

[B63] Serrano R. Kiellandbrandt M. C. Fink G. R. (1986). Yeast plasma-membrane ATPase is essential for growth and has homology with (Na^+^/K^+^), K^+^- and Ca^2+^-ATPases. Nature 319, 689–693 10.1038/319689a0 3005867

[B64] Siglioccolo A. Paiardini A. Piscitelli M. Pascarella S. (2011). Structural adaptation of extreme halophilic proteins through decrease of conserved hydrophobic contact surface. BMC Struct. Biol. 11:50 10.1186/1472-6807-11-50 22192175 PMC3293032

[B65] Srikantha T. Daniels K. J. Pujol C. Sahni N. Yi S. Soll D. R. (2012). Nonsex genes in the mating type locus of Candida albicans play roles in a/alpha biofilm formation, including impermeability and fluconazole resistance. PLoS Pathog. 8:e1002476 10.1371/journal.ppat.1002476 22253594 PMC3257300

[B66] Stanley J. T. Palmer F. Adams J. B. (1982). Microcolonial fungi: common inhabitants on desert rocks? Science 215, 1093–1095 10.1126/science.215.4536.1093 17771840

[B67] Stoop J. M. H. Mooibroek H. (1998). Cloning and characterization of NADP mannitol dehydrogenase cDNA from the button mushroom, *Agaricus bisporus*, and its expression in response to NaCl stress. Appl. Environ. Microbiol. 64, 4689–4696 9835550 10.1128/aem.64.12.4689-4696.1998 PMC90910

[B68] Sun S. Heitman J. (2011). Is sex necessary? BMC Biol. 9:56 10.1186/1741-7007-9-56 21880159 PMC3199587

[B69] Turk M. Plemenitaš A. (2002). The HOG pathway in the halophilic black yeast *Hortaea werneckii*: isolation of the HOG1 homolog gene and activation of HwHog1p. FEMS Microbiol. Lett. 216, 193–199 10.1111/j.1574-6968.2002.tb11435.x 12435502

[B70] Vaupotič T. Plemenitaš A. (2007). Differential gene expression and Hog1 interaction with osmoresponsive genes in the extremely halotolerant black yeast *Hortaea werneckii*. BMC Genomics 8:280 10.1186/1471-2164-8-280 17705830 PMC2034391

[B71] Widmann C. Gibson S. Jarpe M. B. Johnson G. L. (1999). Mitogen-activated protein kinase: conservation of a three-kinase module from yeast to human. Physiol. Rev. 79, 143–180 9922370 10.1152/physrev.1999.79.1.143

[B72] Wollenzien U. Dehoog G. S. Krumbein W. E. Urzi C. (1995). On the isolation of microcolonial fungi occurring on and in marble and other calcareous rocks. Sci. Total Environ. 167, 287–294 10.1016/0048-9697(95)04589-S

[B73] Wosten H. A. (2001). Hydrophobins: multipurpose proteins. Annu. Rev. Microbiol. 55, 625–646 10.1146/annurev.micro.55.1.625 11544369

[B74] Yale J. Bohnert H. J. (2001). Transcript expression in *Saccharomyces cerevisiae* at high salinity. J. Biol. Chem. 276, 15996–16007 10.1074/jbc.M008209200 11278394

[B75] Zajc J. Kogej T. Galinski E. A. Ramos J. Gunde-Cimerman N. (2014). Osmoadaptation strategy of the most halophilic fungus, *Wallemia ichthyophaga*, growing optimally at salinities above 15% NaCl. Appl. Environ. Microbiol. 80, 247–256 10.1128/AEM.02702-13 24162565 PMC3911034

[B76] Zajc J. Liu Y. F. Dai W. K. Yang Z. Y. Hu J. Z. Gostinčar C. (2013). Genome and transcriptome sequencing of the halophilic fungus *Wallemia ichthyophaga*: haloadaptations present and absent. BMC Genomics 14:617 10.1186/1471-2164-14-617 24034603 PMC3849046

[B77] Zalar P. Kocuvan M. A. Plemenitaš A. Gunde-Cimerman N. (2005a). Halophilic black yeasts colonize wood immersed in hypersaline water. Bot. Mar. 48, 323–326 10.1515/BOT.2005.042

[B78] Zalar P. Sybren de Hoog G. Schroers H. J. Frank J. M. Gunde-Cimerman N. (2005b). Taxonomy and phylogeny of the xerophilic genus *Wallemia* (Wallemiomycetes and Wallemiales, cl. et ord. nov.). Anton. Leeuw. 87, 311–328 10.1007/s10482-004-6783-x 15928984

[B79] Zotova L. Aleschko M. Sponder G. Baumgartner R. Reipert S. Prinz M. (2010). Novel components of an active mitochondrial K+/H+ exchange. J. Biol. Chem. 285, 14399–14414 10.1074/jbc.M109.059956 20197279 PMC2863244

